# Predictors of cardiac involvement and survival in patients with primary systemic light-chain amyloidosis: roles of the clinical, chemical, and 3-D speckle tracking echocardiography parameters

**DOI:** 10.1186/s12872-021-01856-3

**Published:** 2021-01-21

**Authors:** Changhui Lei, Xiaoli Zhu, David H. Hsi, Jing Wang, Lei Zuo, Shengjun Ta, Qianli Yang, Lei Xu, Xueli Zhao, Yan Wang, Shiren Sun, Liwen Liu

**Affiliations:** 1 Xijing Hypertrophic Cardiomyopathy Center, Department of Ultrasound, Xijing Hospital, Xi’an, China; 2 Department of Cardiology, Heart and Vascular Institute, Stamford Hospital, Stamford, CT USA; 3 Department of Ultrasound, Yan’an Hospital, Yan’an, Shannxi China; 4 Department of Nephrology, XiJing Hospital, Xi’an, Shannxi China

**Keywords:** Cardiac amyloidosis, Three-dimensional speckle tracking echocardiography, Biomarkers, Strain imaging

## Abstract

**Background:**

Light-chain (AL) amyloidosis is the most common type of systemic amyloidosis with poor prognosis. Currently, the predictors of cardiac involvement and prognostic staging systems are primarily based on conventional echocardiography and serological biomarkers. We used three-dimensional speckle tracking echocardiography (STE-3D) measurements of strain, hypothesizing that it could detect cardiac involvement and aid in prediction of mortality.

**Methods:**

We retrospectively analysed 74 consecutive patients with biopsy-proven AL amyloidosis. Among them, 42 showed possible cardiac involvement and 32 without cardiac involvement. LV global longitudinal strain (GLS), global radial strain, global circumferential strain and global area strain (GAS) measurements were obtained.

**Results:**

The GLS and GAS were considered significant predictors of cardiac involvement. The cut-off values discriminating cardiac involvement were 16.10% for GLS, 32.95% for GAS. During the median follow-up of 12.5 months (interquartile range 4–25 months), 20 (27%) patients died. For the Cox proportional model survival analysis, heart rate, cardiac troponin T, NT-proBNP levels, E/e’, GLS, and GAS were univariate predictors of death. Multivariate Cox model showed that GLS ≤ 14.78% and cardiac troponin T ≥ 0.049 mg/l levels were independent predictors of survival.

**Conclusions:**

STE-3D measurements of LV myocardial mechanics could detect cardiac involvement in patients with AL amyloidosis; GLS and cardiac biomarkers can provided prognostic information for mortality prediction.

## Introduction

Amyloidosis is a disease where the deposition of an amorphous protein-derived substance in the extracellular compartment results in damage to numerous organs. Light-chain (AL) amyloidosis is the most common subtype [1] and is effectively treated with chemotherapy if captured early [2]. However, the diagnosis is often delayed because the clinical symptoms are nonspecific or absent [3]. Cardiac amyloidosis (CA) involves deposition of amyloid fibrils in the myocardial interstitium [4] and portends a particularly poor prognosis. More than half of the patients with AL amyloidosis (51–63%) demonstrate cardiac involvement on diagnosis [5, 6]. Cardiac involvement is the most important prognostic factor in the natural progression of AL amyloidosis that determines prognosis, limits life span [7] and contributes to approximately 75% of deaths [8]. The median survival time is reported to be < 6 months in untreated patients with AL amyloidosis who have cardiac involvement [9].

There have been significant advances in cardiac imaging such as cardiac MRI and nuclear imaging for the diagnosis, risk stratification, amyloid burden and staging of CA. The deposition of amyloid in the heart leads to an increase in myocardial extracellular volume, which is readily detected by CMR through the late gadolinium enhancement (LGE). Nuclear imaging including pyrophosphate imaging aid in the quantification of amyloid load, and 123I-MIBG scintigraphy can detect cardiac sympathetic denervation in amyloidosis patients with cardiac involvement [10, 11]. Recently, speckle-tracking echocardiography-derived myocardial mechanics [12] have demonstrated a potential role in the detection of cardiac involvement and in the prediction of prognosis in patients with amyloidosis [13]. Three-dimensional speckle-tracking echocardiography (STE-3D) is a technique with the potential for non-invasive assessment to track myocardial motion in the three-dimensional space [14].

The current study aimed to examine left ventricular (LV) myocardial strain in patients with AL amyloidosis by STE-3D to detect cardiac involvement, predict mortality, and to define the cut-off points for cardiac involvement in this population.

## Methods

We retrospectively analysed 74 consecutive patients who were newly diagnosed with immunoglobulin AL amyloidosis and referred to XiJing Hospital, Xi'an, China, for echocardiographic studies from October 22, 2014, through January 4, 2018. The diagnosis of immunoglobulin AL amyloidosis was made by histological confirmation of AL amyloid deposits in involved organs (at least one biopsy specimen from either endomyocardial tissue, bone marrow, kidney, liver or nerve). The histologic diagnostic criteria were Congo red-positive deposition on light microscopy and nonbranching fibrils 8–10 nm in diameter on electron microscopy. Immunohistochemical staining confirmed single kappa or lambda light chain positivity. Immunofixation electrophoresis of blood and serum free light-chains also provided evidence of a monoclonal protein.

**Fig. 1:**
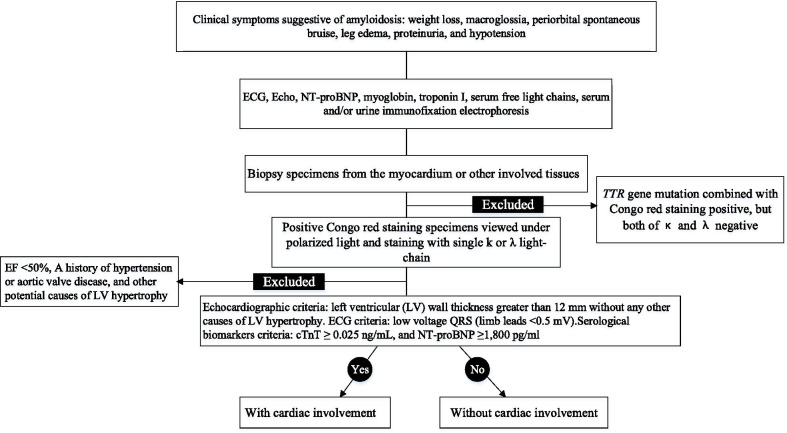
Study design and group classification

We mainly used key ECG and echocardiographic criteria for CA in patients with confirmed light-chain immunoglobulin AL amyloidosis such as mismatches of low voltage ECG, and LVH on echo with a meanLV wall thickness greater than 12 mm without any other causes of LV hypertrophy, plus supportive evidence of cardiac involvement including elevated troponin and pro-BNP levels [15–17]. The flowchart in Fig. 1 demonstrated our diagnostic algorithm.

Patients with MTTR amyloidosis diagnosed by identification of *TTR* gene mutation combined with Congo red staining were not included in the study. Patients with left ventricular ejection fraction (LVEF) < 50%, a history of hypertension or aortic valve disease, and other potential causes of LV hypertrophy were identified before enrolment and were excluded from the study.

Patients were categorized into stages I through IV based on scores according to the revised 4-point Mayo staging system [17]. A score of 1 was assigned for each of the following variables: troponin I ≥ 0.1 ng/mL, N-terminal pro-B-type natriuretic peptide (NT-proBNP) ≥ 1800 pg/mL, Free light chain difference (FLC-diff) ≥ 18 mg/dL.

Patients’ demographic characteristics, clinical data, electrocardiography and tissue biopsy results were obtained from hospital records. Echocardiographic studies were performed at the time of diagnosis for all patients. There was a maximum time interval of < 2 weeks between obtaining echo studies and performing biopsies. Patients were followed with the endpoint of all-cause mortality. Follow-up was obtained by reviewing the patients’ medical records or by telephone interviews with the patients or relatives and was completed before December 30, 2018. The study was performed in accordance with the principles of the Declaration of Helsinki and was approved by the institutional ethics committee. All patients were provided with a written informed consent by the department of nephrology or cardiology during they hospitalization at XiJing Hospital. We followed a strict institutional protocol regarding diagnosis, initial evaluation, treatment, and retrospective observation in this patient cohort.

### Conventional echocardiography measurements

All echocardiographic studies were performed using an iE33 ultrasound system (Philips Medical Systems, Bothell, WA, USA) with a 1.0–5.0 MHz broad-band transducer. The subjects were placed in the left lateral recumbent position, and the ECG was recorded simultaneously. Conventional echocardiography was performed with an S5-1 probe. The maximum LV wall thickness (MLVWT). LV end-diastolic volume (LVEDV), LV end-systolic volume (LVESV), left atrial volume (LAV), LVEF, peak early filling (E-wave) and late diastolic filling (A-wave) velocities was measured according to the standardized American Society of Echocardiography protocol [18]. The E/A ratio was calculated. LAV were indexed to body surface area [19]. The early diastolic peak velocities (e’) were measured at the septal and lateral insertion sites of the mitral leaflets and averaged e’ as an index of LV relaxation. The E/e’ ratio was calculated as an index of LV filling pressures. We did not use left ventricular mass index for the predictive model since it might be secondary to the changes involving cardiac amyloidosis.

### Three-dimensional STE strain measurements

An X5-1 matrix-array transducer (Philips Medical Systems, Bothell, WA, USA) was used to acquire full-volume 3D images from the apical four-chamber views. The images were adjusted by biplane mode to obtain images that included the entire LV with clear endocardial borders. The participants were asked to hold their breath during full-volume 3D data acquisition. The off-line analysis was performed by transferring full-volume 3D images to a TomTec workstation using analysis software (4D LV-analysis 3.2; TomTec Imaging Systems GmbH, Bayern, Germany). Apical two-chamber, three-chamber and four-chamber views were automatically selected at end-diastole.

Once the user confirmed that the automatically selected views and the image frames were correct, the algorithm automatically identified the LV endocardial boundary at end-diastole. After this step, the reader could manually edit the boundaries. Subsequently, the LV was divided into 16 segments using standard segmentation and expressed as per segment, per level (basal, mid, or apical) mean, or global (all segments) mean. The 3D strain measurements derived included LV peak systolic global longitudinal strain (GLS), global radial strain (GRS), global circumferential strain (GCS), and global area strain (GAS), which is defined as the combination of GLS and GCS. GLS, GRS, and GCS were averaged over the 16 segments. Segment longitudinal strain included: basal, medial, and apical segment, apical to basal ratio (apical segments divided by basal segments), relative apical sparing (apical and mid segments divided by basal segments). The peak value of each strain measurement was defined as its maximum absolute value with a positive sign. The observer was blinded to cardiac involvement or survival status.

### Biomarkers

In all patients with AL amyloidosis, blood was collected for the measurement of the levels of myoglobin, troponin I, N-terminal proB-type natriuretic peptide (NT-proBNP), cardiac troponin T, serum and/or urine kappa lambda protein, and creatinine. Estimated glomerular filtration rate (eGFR) was calculated by CKD-EPI formula. The mean difference between kappa or lambda free-light chain (FLC) in the involved FLC and uninvolved FLC was reported as FLC-diff. Protein levels were measured from the 24-h urine collection.

### Intra-observer and inter-observer variability analysis

Intra-observer and interobserver variability was assessed using the intraclass correlation coefficient. A *P* value < 0.05 was considered to indicate statistical significance. Reproducibility statistical analyses were performed using MedCalc statistical software version 15.2.2 (MedCalc Software, Ostend, Belgium). Intra-observer variability was calculated by repeated measurements by the primary reviewer (CHL) 2 months after the initial measurement. Interobserver variability was calculated by comparing the original GLS and GAS calculation with that calculated by a blinded second observer (XLZ1).

### Statistical analysis

Continuous variables were expressed as mean ± SD or median IQR, and categorical variables as frequencies and percentages. The continuous data were compared using the Student’s *t* tests and Wilcox on-rank sum tests when assumptions were not met. For the categorical variables, either Pearson’s chi-squared or Fisher’s exact tests were used. We studied echo parameters and STE-3D between the Cardiac Involvement (CI) and without CI. Based on the univariate analysis, clinically relevant parameters were grouped for multivariate logistic regression model by incorporating a set of variables that were most statistically significant and with potential clinical relevance.

For the entire cohort, Kaplan–Meier analysis was used to estimate overall survival, and differences between groups were tested for statistical significance using the 2-tailed log-rank test. Overall survival was defined as the time from the date of initial diagnosis of AL to the date of death or last follow-up. In order to identify risk factors for mortality. Univariate cox proportional hazards regression analyses were used for each of the variables, Multivariate Cox regression analysis was performed by incorporating into the model a set of variables that were most statistically significant according to univariate analysis or based on their potential clinical relevance. All the statistical analyses were performed using SPSS statistical software (Version 17; SPSS Inc, Chicago, IL, USA) and MedCalc statistical software version 15.2.2 (MedCalc Software, Ostend, Belgium). *P* value < 0.05 was considered statistically significant.

## Results

### Baseline clinical characteristics and serological biomarker evaluations

Total of 74 patients with AL amyloidosis were included (mean age, 57.6 ± 10.0 years, 51.4% males): 42 (57%) with cardiac involvement (CI) and 32 (43%) without cardiac involvement (CI). Seventeen patients (40.5%) with cardiac involvement and three patients (9.4%) without cardiac involvement died during the follow-up. Patients’ baseline clinical characteristics and serological biomarker data are summarised in Table 1. Patients with cardiac involvement were more likely to had higher troponin I, cardiac troponin T and NT-proBNP levels. Mayo cardiac stage was significantly associated with cardiac involvement (*P* < 0.001), For the entire study population, all 32 patients without cardiac involvement were of Mayo stage I and II. Among 42 patients with cardiac involvement, 11 (26.2%) patients were with Mayo stage I and II, while 31(73.8%) patients were with Mayo stage III and IV.

**Table 1:** Clinical and biomarkers characteristics in amyloidosis patients by cardiac involvement (CI) and survival status

Parameters	All patients (n = 74)	With CI (n = 42)	Without CI (n = 32)	*P* value	Non-survivors (n = 20)	Survivors (n = 54)	*P* value
Age (years)	57.6 ± 10.0	57.8 ± 10.2	57.2 ± 9.9	0.778	57.0 ± 11.4	57.78 ± 9.5	0.769
Male (%)	38 (51.4)	25 (59.5)	13 (40.6)	0.107	12 (60)	26 (48.1)	0.365
BSA (m^2^)	1.68 ± 0.15	1.68 ± 0.17	1.68 ± 0.12	0.938	1.66 ± 0.17	1.68 ± 0.14	0.599
SBP (mm Hg)	116 ± 17	114 ± 18	119 ± 16	0.211	109 ± 15	119 ± 17	0.049
DBP (mm Hg)	73 ± 12	71 ± 9	75 ± 14	0.226	69 ± 10	74 ± 12	0.102
HR (beats/min)	81 ± 15	83 ± 15	78 ± 15	0.129	88 ± 13	78 ± 15	0.012
Mayo stage							
I and II (%)	43 (58.1)	11 (26.2)	32 (100)	< 0.001	7 (35)	36 (66.7)	0.014
III and IV (%)	31 (41.9)	31 (73.8)	0 (0)		13 (65)	18 (33.3)	
Troponin I (ng/mL)	0.04 (0.15)	0.10 (0.27)	0.01 (0.02)	< 0.001	0.15 (0.41)	0.025 (0.07)	0.003
cTnT (ng/mL)	0.03 (0.06)	0.06 (0.11)	0.02 (0.02)	< 0.001	0.10 (0.14)	0.03 (0.04)	0.001
NT-proBNP (pg/ml)	769 (2901)	2436 (6514)	348 (476)	< 0.001	4062 (8126)	504 (1578)	< 0.001
FLC-diff(mg/L)	45.0 (88.2)	75.7 (106.3)	27.3 (57.4)	0.111	100.8 (82.9)	23.9 (87.6)	0.094
Serum creatinine (μmol/L)	88.0 (32)	92.5 (39.3)	84.5 (22.3)	0.194	93.5 (56.3)	86.5 (29.8)	0.139
eGFR (ml/min/1.73m^2^)	69.4 ± 23.7	74.0 ± 25.9	66.0 ± 21.6	0.154	61.6 ± 21.6	72.4 ± 24.1	0.083
24 h urinary protein (g/day)	2.27 (2.23)	2.33 (2.75)	2.27 (2.06)	0.966	1.95 (3.19)	2.34 (2.11)	0.904

*BSA* body surface area, *SBP* systolic blood pressure, *DBP* diastolic blood pressure, *HR* heart rate, *(FLC-diff)* free light chain differential, *eGFR* estimated glomerular filtration rate

### Conventional echocardiography parameters

Conventional echocardiographic parameters are detailed in Table 2. Patients with cardiac involvement had thicker walls, larger left atrium volume index, lower mean early diastolic tissue Doppler velocity at the septal and lateral mitral annulus and increased mean E/e’ ratio of mitral inflow than patients without cardiac involvement.

**Table 2:** Conventional echocardiography parameters in amyloidosis patients by cardiac involvement and survival status

Parameters	All patients (n = 74)	With CI (n = 42)	Without CI (n = 32)	*P* value	Non-survivors (n = 20)	Survivors (n = 54)	*P* value
MLVWT (mm)	13.05 ± 3.01	15.17 ± 2.23	10.26 ± 0.75	< 0.001	15.47 ± 2.91	12.15 ± 2.53	< 0.001
LVEDV (ml)	79.27 ± 27.20	81.90 ± 32.25	75.81 ± 18.57	0.343	74.50 ± 21.58	81.04 ± 28.99	0.362
LVESV (ml)	33.48 ± 18.16	35.61 ± 22.77	30.69 ± 8.75	0.251	32.83 ± 11.44	33.72 ± 20.17	0.852
LVEF (%)	58.56 ± 6.02	57.75 ± 6.96	59.62 ± 4.39	0.187	56.23 ± 5.10	59.42 ± 6.15	0.042
LAVI (ml/m^2^)	31.79 ± 10.52	35.61 ± 11.10	26.79 ± 7.21	< 0.001	34.97 ± 14.54	30.52 ± 8.34	0.208
E/A	1.22 ± 1.44	1.21 ± 0.75	1.25 ± 2.02	0.909	1.29 ± 0.85	1.20 ± 1.60	0.815
e’ (cm/s)	6.11 ± 2.16	5.05 ± 1.69	7.51 ± 1.91	< 0.001	4.58 ± 1.80	6.68 ± 2.00	< 0.001
E/e’	13.24 ± 7.18	16.57 ± 7.69	8.96 ± 3.15	< 0.001	17.77 ± 8.85	11.31 ± 5.81	< 0.001

*MLVWT* maximum left ventricular wall thickness, *LVEDV* left ventricular end-diastolic volume, *LVESV* left ventricular end-systolic volume, *LAVI* left atrial volume index, *LVEF* left ventricular ejection fraction, *E* early diastolic mitral flow velocity, *A* late diastolic mitral flow velocity, *e′* early diastolic tissue Doppler velocity at medial mitral annulus

### Three-dimensional speckle tracking echocardiography-derived parameters

**Fig. 2:**
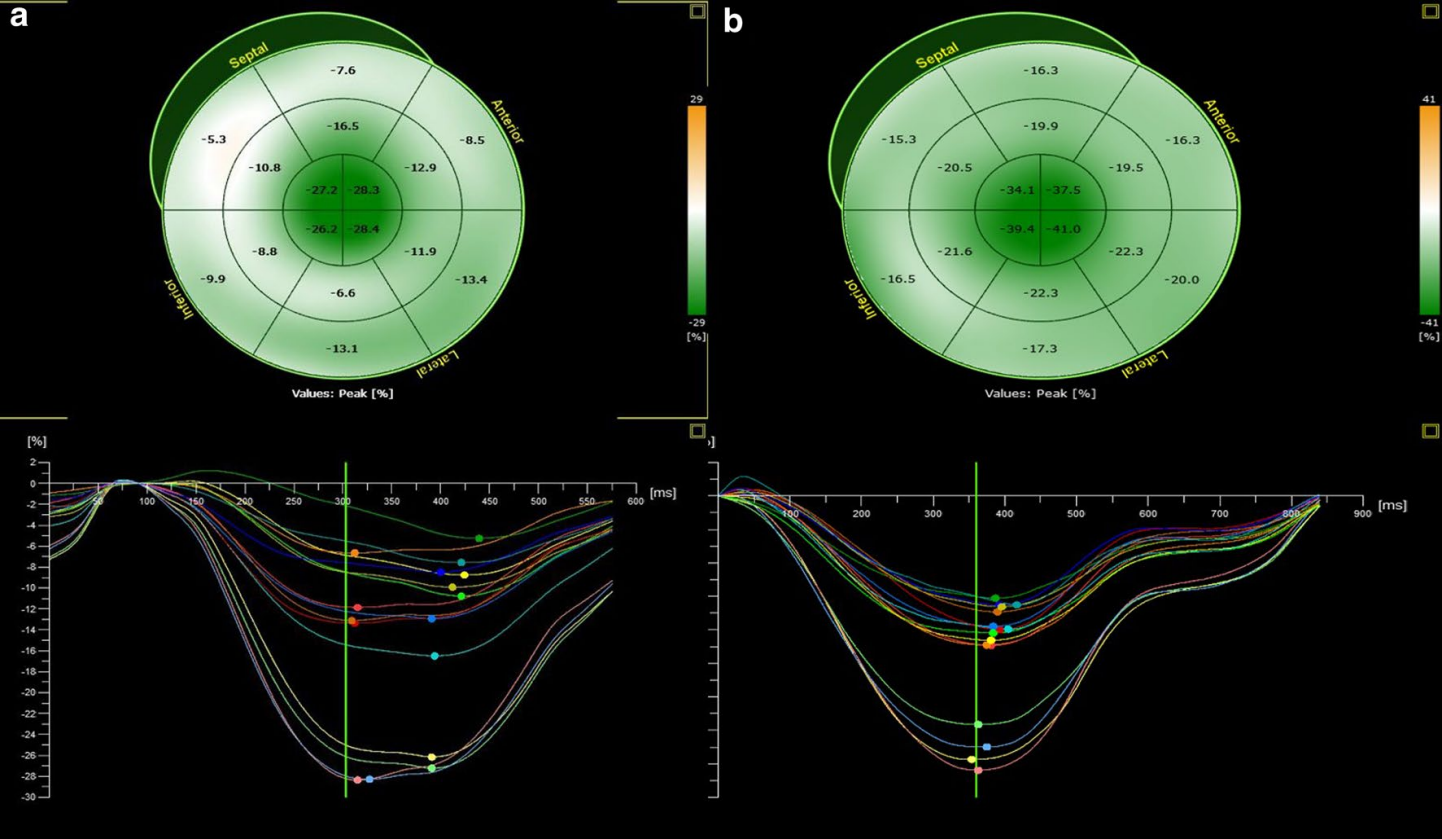
Longitudinal strain curve of amyloidosis patients with and without cardiac involvement. Note the separation of strain curves with higher strain in the apical versus basal segments as well as the prominent green color indicating higher strains in the apical segments STE-3D maps. **a** Longitudinal strain curve of patients with cardiac involvement. **b** Longitudinal strain curve of patients without cardiac involvement

Three-dimensional speckle tracking echocardiography parameters are featured in Table 3. LV longitudinal, radial and area strain were reduced in patients with cardiac involvement compared to patients without cardiac involvement with the most prominent impairment at the basal and medial segments. GLS, GAS, and GRS were significantly lower in patients with cardiac involvement; Ratios of regional strain demonstrated an increase in apical relative to basal strain, and relative apical sparing as illustrated by the case example in Fig. 2.

**Table 3:** Three-dimensional speckle tracking echocardiography-derived parameters in amyloidosis patients by cardiac involvement and survival status

Parameters	All patients (n = 74)	With CI (n = 42)	Without CI (n = 32)	*P* value	Non-survivors (n = 20)	Survivors (n = 54)	*P* value
Global strain							
GLS (%)	15.79 ± 2.37	14.28 ± 1.89	17.76 ± 1.14	< 0.001	14.22 ± 2.68	16.37 ± 1.96	< 0.001
GRS (%)	36.35 ± 3.70	34.64 ± 3.72	38.60 ± 2.19	< 0.001	34.32 ± 3.85	37.10 ± 3.38	0.003
GCS (%)	27.97 ± 2.94	27.59 ± 3.30	28.49 ± 2.33	0.196	27.32 ± 2.52	28.22 ± 3.06	0.240
GAS (%)	32.45 ± 3.01	31.62 ± 3.28	33.56 ± 2.27	0.005	31.17 ± 2.97	32.93 ± 2.93	0.025
Segments longitudinal strain							
Basal segments(%)	15.51 ± 3.89	13.35 ± 3.09	18.33 ± 2.94	< 0.001	12.30 ± 3.51	16.69 ± 3.34	< 0.001
Medial segments (%)	14.54 ± 2.59	13.32 ± 2.16	16.15 ± 2.22	< 0.001	13.79 ± 2.71	14.82 ± 2.51	0.132
Apical segments (%)	18.78 ± 4.05	17.89 ± 3.94	19.96 ± 3.95	0.029	18.61 ± 5.12	18.85 ± 3.64	0.823
Apical/basal ratio	1.31 ± 0.51	1.44 ± 0.56	1.14 ± 0.36	0.006	1.62 ± 0.62	1.19 ± 0.40	0.009
Relative apical sparing	2.29 ± 0.72	2.48 ± 0.79	2.05 ± 0.55	0.007	2.78 ± 0.82	2.11 ± 0.59	0.002

*GLS* global longitudinal strain, *GRS* global radial strain, *GCS* global circumferential strain, *GAS* global area strain

### Optimal cut-off points of STE-3D parameters for cardiac involvement

The results of the univariate and multivariate regression analyses of predictors of cardiac involvement in patients with AL amyloidosis are demonstrated in Table 4. The STE-3D parameters GLS, GAS were significant predictors of cardiac involvement in AL amyloidosis, with GLS showing the highest odds ratio of 4.713.

**Table 4:** Univariate regression analysis of predictors cardiac involvement in AL amyloidosis patients

Parameters	Univariate OR (95% CI)	*P* value	Multivariate OR (95% CI)	*P* value
Age (years)	1.007 (0.961–1.055)	0.774	NI	NA
Gender	0.465 (0.182–1.187)	0.109	NI	NA
Log cTnT (mg/l)	21.22 (4.514–99.71)	< 0.001	NI	NA
LogNT-proBNP (pg/ml)	11.30 (3.683–34.682)	< 0.001	2.386 (1.081–5.265)	0.031
LAVI (ml/m^2^)	1.1008 (1.042–1.177)	0.001	NI	NA
E/e’	1.358 (1.159–1.591)	< 0.001	0.020 (1.035–1.006)	0.020
GLS (%)	4.516 (2.410–8.462)	0.000	1.310 (1.050–1.632)	0.016
GRS (%)	0.554 (0.414–0.742)	0.000	NI	NA
GAS (%)	1.318 (1.072–1.621)	0.009	1.126 (1.020–1.244)	0.019

*NI* not included in model, *NA* not applicable

**Fig. 3:**
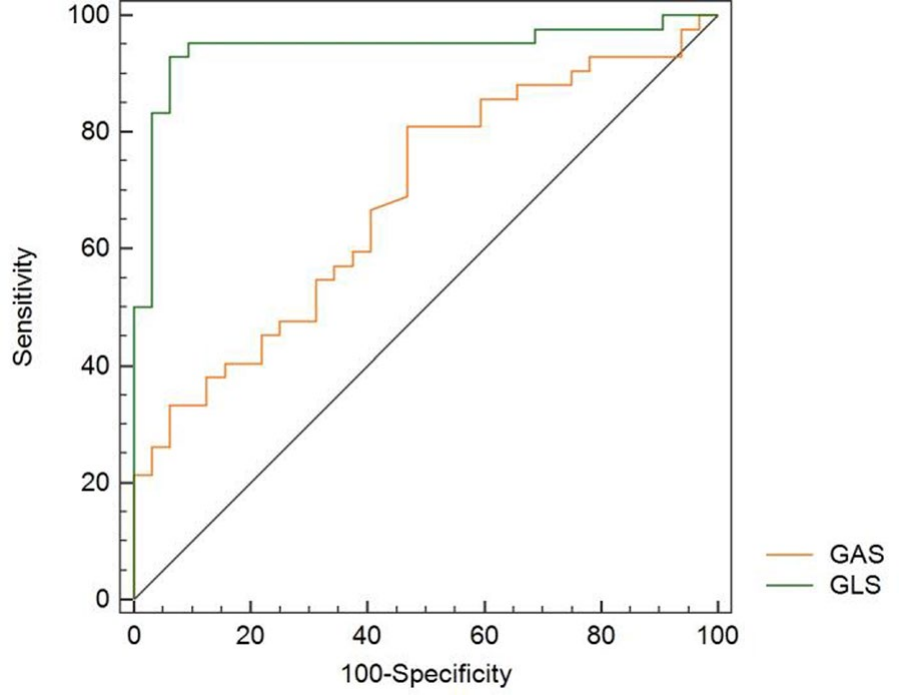
ROC curves comparing STE-3D echocardiographic parameters for their accuracy to predict cardiac involvement

ROC analyses were performed to determine the optimal thresholds associated with cardiac involvement (Fig. 3). A baseline GLS of 16.10% predicted cardiac involvement with sensitivity of 92.9% and specificity of 93.7%, AUC 0.943. A baseline GAS ≤ 32.95% predicted cardiac involvement with a sensitivity of 81% and specificity of 53.1%, AUC 0.68, weaker than that of GLS.

### Intra-observer and inter-observer variability analysis

For GLS, the intra-class correlation coefficient for intra-observer agreement was 0.95 (95% CI, 0.905–0.972); and the inter-observer agreement was 0.90 (95% CI, 0.796–0.955). For GAS, the intra-class correlation coefficient for intra-observer agreement was 0.93 (95% CI, 0.844–0.973); and the interobserver agreement was 0.87 (95% CI, 0.713–0.944), reflecting substantial agreement for measurement of GLS and GAS.

### Survival analysis

**Fig. 4:**
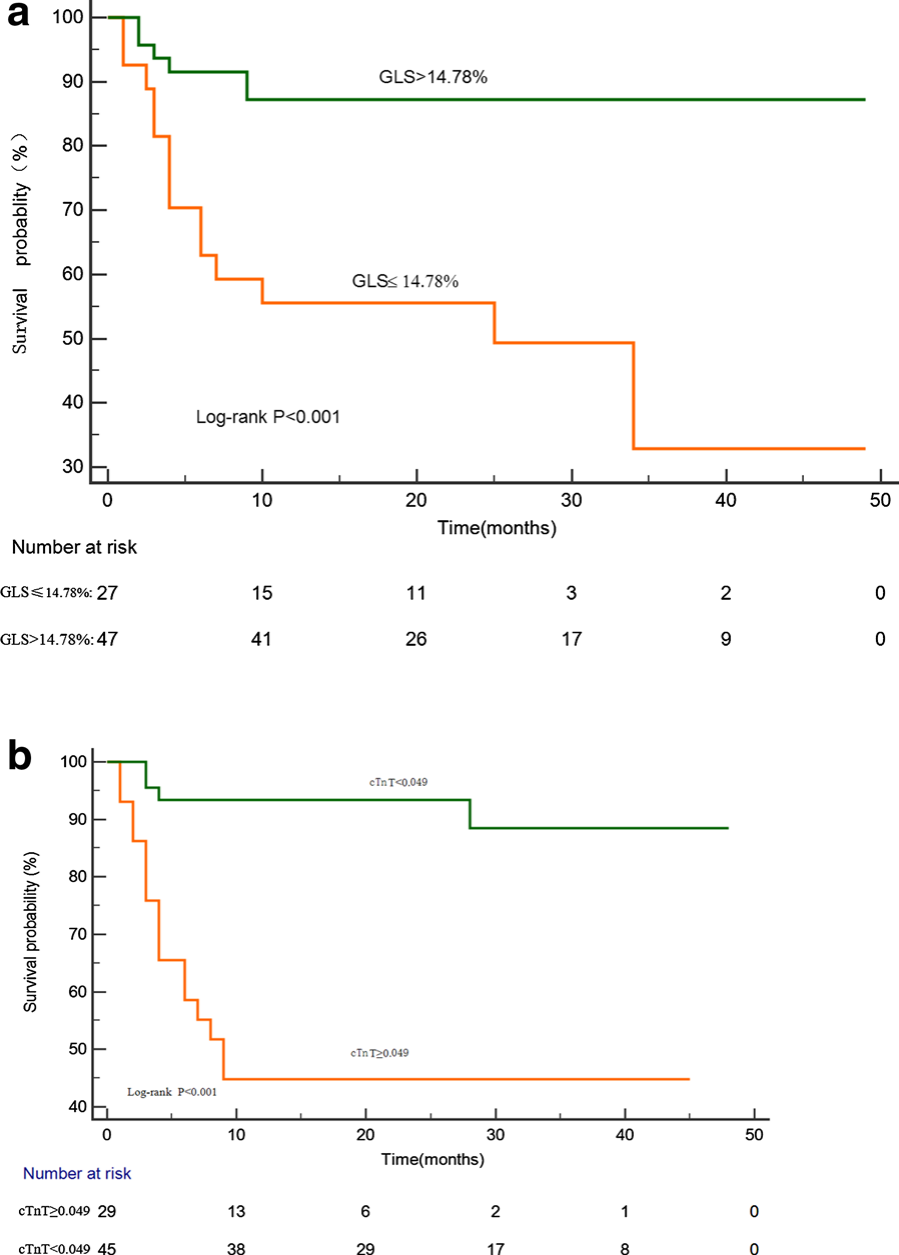
Kaplan–Meier representation of survival as stratified by GLS and cTnT. Patients with GLS baseline lower than 14.78% had a significant reduction in survival (**a**). Patients with cTnT baseline higher than 0.049 had a significant reduction in survival (**b**)

During the median follow-up of 12.5 months (range 4–25 months), 20 (27%) patients with AL amyloidosis died, 9 died due to sudden death, 1 due to cerebral haemorrhage, 6 due to heart failure, 1 because of renal failure, 1 after gastrointestinal bleeding, and, 2 due to multi-organ failure. Tables 1, 2 and 3 illustrated the baseline clinical characteristics and echocardiographic measurements of the survivors (n = 54) and non-survivors (n = 20). Among the echocardiographic parameters, MLVWT, mean E/e’ ratio, GLS, GRS, GAS, apical/basal ratio and relative apical sparing showed significant differences (*P* < 0.05) between survivors and non-survivors. The univariable Cox regression survival analysis is shown in Table 5. Heart rate, cTnT, NT-proBNP levels, E/e’, GLS and GAS were univariate predictors of all-cause mortality. In the multivariate survival analysis GLS ≤ 14.78% (HR: 1.275; 95% CI: 1.017 to 1.597), and the biomarkers cTnT ≥ 0.049 mg/l (HR: 5.552; 95% CI: 1.543 to 19.981) was an independent predictor of survival. GLS ≤ 14.78% and cTnT ≥ 0.049 mg/l provided the best cut-off values to predict death (*p* < 0.001, respectively) (Fig. 4a, b).

**Table 5:** Univariate and multivariate Cox proportional models of predictors of survival

Parameters	Univariate HR (95% CI)	*P* value	Multivariate HR (95% CI)	*P* value
Age (years)	0.995 (0.952–1.039)	0.796	NI	NA
HR (beats/min)	1.036 (1.008–1.066)	0.011	NI	NA
Log cTnT (ng/mL)	8.659 (2.847–26.337)	< 0.001	5.552 (1.543–19.981)	0.009
Log NT-proBNP (pg/ml)	3.596 (1.696–7.512)	0.001	NI	NA
E/e’	1.086 (1.036–1.139)	0.001	1.142 (0.891–1.463)	0.294
GLS ≤ 14.78%	1.385 (1.158–1.656)	< 0.001	1.275 (1.017–1.597)	0.035
GAS (%)	1.150 (1.031–1.284)	0.013	NI	NA

*NI* not included in model, *NA* not applicable

## Discussion

This study comprehensively assessed cardiac properties of patients with AL amyloidosis analysing clinical, conventional echocardiography, serological biomarker and STE-3D derived myocardial function data. The primary findings of the study were as follows: GLS and GAS were significantly decreased in patients with cardiac involvement. The most prominent decrease was at the basal and medial segments, as manifested by the apical to basal strain ratio and relative apical sparing. GLS threshold ≤ 16.10% and GAS threshold ≤ 32.95% demonstrated predictive value in patients with AL amyloidosis. Abnormalities in GLS, GAS, apical/basal ratio and relative apical sparing assessed with STE-3D were also associated with reduced survival.

AL amyloidosis is characterised by a relatively low burden of clonal plasma cells and involvement of multiple organs by immunoglobulin light chain-derived amyloid fibrils [20]. The course of the disease is more likely to be progressive if there is cardiac involvement [7, 21]. Cardiac involvement is the main cause of death in patients with AL amyloidosis, and is an important prognostic factor. Conventional echocardiographic features typically include characteristic thickening of the left and right ventricular wall, valvular thickening, a sparkling texture of the myocardium, pericardial effusion and advanced diastolic dysfunction [22, 23]. The degree of LV hypertrophy is associated with poor outcomes in these patients [24]. Previous findings showed that longitudinal strain abnormalities were evident before the conventional echocardiography [25, 26].

Koyama et al. [27] demonstrated the ability of longitudinal strain to identify cardiac involvement in systemic AL amyloidosis with a TDI-derived strain. Several previous studies on AL amyloidosis have shown that the longitudinal strain determination by STE-2D was effective in detecting early cardiac involvement [15, 28]. Barros-Gomes et al. [15] observed that the longitudinal strain showed significant differences between cardiac and non-cardiac involvement. In recent years, STE-3D was developed on the basis of real-time three-dimensional echocardiography and speckle tracking technology. It has now emerged as a very useful method for analysing the complexity of LV mechanics in the three-dimensional space [29]. STE-3D offers additional deformation parameters (such as area strain) and a comprehensive quantification of LV geometry and function from a single 3D acquisition. STE-3D has provided new insights into LV mechanics in several clinical fields, such as the assessment of global and regional LV function in coronary artery disease [30], valvular heart disease [31] and examination of left and right ventricular myocardial mechanics in light-chain CA [21].

Our study demonstrated the value of STE-3D parameters in identifying cardiac involvement in systemic AL amyloidosis. We found that patients with cardiac amyloidosis presented with considerable impairment in LV mechanics. They had decreased GLS, GAS, increased apical/basal ratio and relative apical sparing values. Our findings confirmed other published reports of longitudinal strain to predict cardiac involvement in patients with AL amyloidosis. LV longitudinal strain was markedly decreased in cardiac amyloidosis patients with the most prominent reduction in the basal segments while the apical strain was preserved. Basal strain impairment, relative to apical strain preservation, has been reported and serves as a useful discriminator between the AL-CA and other forms of myocardial hypertrophy such as systemic arterial hypertension, athletes and hypertrophic cardiomyopathy [32]. Based on our data, we calculated cardiac involvement threshold of GLS ≤ 16.10%, with a high sensitivity of 92.9% and specificity of 93.7%.

In AL amyloidosis, GLS abnormalities were likely related to immunoglobulin deposition in the LV endocardium. Cardiac amyloid burden can be reflected by myocardial extracellular volume fraction [33]. The motion along the long axis of the heart is probably the most fundamental mechanism of the ventricles twisting and untwisting in combination with longitudinal shortening [34]. Longitudinal LV mechanics are the most sensitive for early changes in myocardial disease. Magnetic resonance tomographic observations also demonstrated that the subendocardial myocardium was affected first by the disease [35], supporting the notion that longitudinal fibers were involved during the early disease course.

Global area strain (GAS) reflects the relative area of change that combines of both longitudinal and circumferential shortening [29]. Given that GAS results from the combination of longitudinal and circumferential strain [36], making it attractive for the study of LV subclinical dysfunction. In our study population, reduced GAS was an independent predictor of cardiac involvement in AL amyloidosis; however, its diagnostic accuracy was weaker than that of GLS for the early detection of LV myocardium impairment. Nevertheless, based on the multivariate analysis it may have an incremental value for predicting survival in our patients with AL amyloidosis. In the current study, non-survivors had a significant decrease in GLS and GAS in both univariate and multivariate analyses. In the multivariate Cox model, a GLS less than 14.78% was an independent predictor of survival. Our observation underscored the potential benefit of strain for predicting outcome. Previous studies have reported that reduced LV longitudinal strain function was an independent predictor of survival in AL amyloidosis [15, 26, 37, 38]. Buss et al. [38] demonstrated that quantification of LV longitudinal function by LS and 2D-GLS provided incremental prognostic value regarding the cardiovascular outcomes in AL amyloidosis and that this approach appeared to be superior to standard echocardiography. Barros-Gomes et al. [15] demonstrated that GLS ≥ − 14.81% was independent predictors of survival and incorporation of GLS into the echocardiographic examination of patients with AL amyloidosis improves the risk stratification for survival. We do want to point out that myocardial biomarkers including cardiac troponin levels remain as the most important survival predictors, while measurement of the LV myocardial mechanics of GLS assessed by STE-3D contributed to the prediction of survival in patients with AL.

We feel that multi-modality imaging will play important roles for the diagnosis of AL amyloidosis with cardiac involvement. A recently published large European study used T1 mapping for the assessment of CA. A native T1 < 1036 ms was associated with 98% negative predictive value for CA whereas a native T1 > 1164 ms was associated with 98% positive predictive value for CA. The authors proposed using these cut-off values to exclude or confirm CA and to restrict the administration of contrast only to patients with intermediate probability [39]. Preliminary works by Moñivas et al. [40] suggested that free wall RV strain showed same apical sparing pattern as the left ventricle, but basal and mid involvement varied among different subtypes. By combing echo, nuclear, cardiac MRI imaging, and serum biomarkers, clinicians will be able to diagnose patients with cardiac amyloidosis, and provide accurate assessment of the clinical staging, amyloid burden, and prognosis.

## Study limitations

Our study is a retrospective, single-center cohort with a relatively small sample size. In future studies, a sufficiently large number of patients with confirmed cardiac events will be important to verify the results of our survival analyses and to explore the value of other strain parameters. A single diagnostic gold standard such as cardiac biopsy was not used in all patients to define cardiac involvement. The reference values may vary among studies due to the use of different scanners and software algorithms for computing 3D strain. The STE-3D parameters GLS and GAS are not standardized yet, and the cut-off values identified in the current study might not be applicable when using a system different from our study. We did not have RV STE-3D data in our patients. Once validated in the future, RV STE-3D may potentially be utilized to assess the mechanics of the right ventricle and provide additional information for assessing prognosis in CA patients.

## Conclusions

The prognosis was poor for patients with AL amyloidosis and cardiac involvement. Early identification of patients with cardiac involvement is essential. Our data suggest that STE-3D measurement of the LV myocardial mechanics could identify early cardiac impairment and improve risk stratification in patients with primary systemic AL amyloidosis, and provide incremental benefit beyond standard assessments such as 2D echocardiographic metrics, troponin T and NT-proBNP.

## Data Availability

The datasets used and/or analysed during the current study are available from the corresponding author on reasonable request.

## Abbreviations

AL: Light-chain
CA: Cardiac amyloidosis
BSA: Body surface area
SBP: Systolic blood pressure
DBP: Diastolic blood pressure
HR: Heart rate
LV: Left ventricular
FLC-diff: Free light chain differential
STE-3D: Three-dimensional speckle-tracking echocardiography
TDI: Tissue Doppler imaging
MLVWT: Maximum left ventricular wall thickness
LVEDV: Left ventricular end-diastolic volume
LVESV: Left ventricular end systolic volume
LAVI: Left atrial volume index
LVEF: Left ventricular ejection fraction
E: Early diastolic mitral flow velocity
A: Late diastolic mitral flow velocity
e’: Early diastolic tissue Doppler velocity at medial mitral annulus
GLS: Global longitudinal strain
GRS: Global radial strain
GCS: Global circumferential strain
GAS: Global area strain
eGFR: Estimated glomerular filtration rate
CI: Cardiac involvement
